# Treatment-related adverse events as surrogate to response rate to immune checkpoint blockade

**DOI:** 10.1097/MD.0000000000022153

**Published:** 2020-09-11

**Authors:** Yanyun Shen, Yunfeng Chen, Duoqin Wang, Zhidong Zhu

**Affiliations:** a Department of Dermatology; b Department of Anesthesia; c Department of Cardiology, Huashan Hospital, Fudan University, PR China.

**Keywords:** adverse events, cancer immunity, immune checkpoint blockade

## Abstract

**Background:**

Immune checkpoint blockade (ICB) brings hope to many late-stage cancer patients yet its marker for response remains elusive.

**Methods:**

We developed a hypothesis that treatment-related adverse events (TrAEs) could predict objective response rate (ORR) to ICB. We plotted ORR against corresponding any and grade 3 to 5 (G3–5) TrAEs across a variety of cancer types by performing a meta-analysis using linear regression.

**Results:**

We identified 113 eligible studies encompassing 25 types of malignancies that were treated with ICB or ICB-based regimes. A significant linear correlation was observed for any and severe TrAEs, respectively. The correlation coefficient was 0.57 (*r*^2^ = 0.324) for any TrAE and 0.61 (*r*^2^ = 0.37) for G3–5 TrAE. For melanoma, the correlation coefficient was 0.81 (*r*^2^ = 0.57) for any TrAE and 0.65 (*r*^2^ = 0.42) for G3–5 TrAEs. For RCC, the correlation coefficient was 0.86 (*r*^2^ = 0.74) for any TrAE and 0.91 (*r*^2^ = 0.83) for G3–5 TrAE. For NSCLC, the correlation coefficient was 0.55 (*r*^2^ = 0.3) for any TrAE and 0.74 (*r*^2^ = 0.86) for G3–5 TrAE. For UC, the correlation coefficient was 0.47 (*r*^2^ = 0.68) for any TrAE and 0.27 (*r*^2^ = 0.52) for G3–5 TrAE, yet the correlation was insignificant for severe AEs.

**Conclusion:**

Our findings suggest that over half of ICB responses could be reflected by any adverse events and ∼60% of responses could be reflected by severe AEs. Further validation is needed in individual trials.

## Abstract 2

Supplemental Digital Content is available in the text

## 1 Introduction

Immune checkpoint blockade (ICB) brings hope to late-stage cancer patients as its emergence in recent years altered treatment guidelines of many cancers drastically.^[1]^ Currently there are 6 FDA-approved monoclonal antibodies including Nivolumab, Atezolizumab, Durvalumab, Pembrolizumab, Avelumab, and Ipilimumab that target programmed cell death-1 (PD-1) and its ligand (PD-L1), and cytotoxic T-lymphocyte antigen 4 (CTLA-4). Monotherapy or combination therapy with other targeted medications have now been upgraded to the frontline therapy in advanced stage of several types of cancer, like renal cell carcinoma (RCC)^[2,3]^ and urothelial carcinoma.^[4,5]^

Nevertheless, efficacy of ICB shows substantial polarization. While in responders ICB shows satisfactory and durable effect, the objective response rate (ORR) across all cancer types tested in trial is roughly ∼26%. Given its potential toxicity^[6]^ and inferior cost-effectiveness,^[6,7]^ clinical markers for potential responders are at urgent need. Thus far, only some specified immunohistochemical staining of PD-L1 in certain cancer types matched to specific ICB drug are approved by FDA.^[8]^ However, access to such diagnostic tests is variable and limited at many institutions.

Recently, association has been reported between immune-related adverse events (IrAEs) and response to ICB in melanoma.^[9]^ Whereas multiple studies concerning melanoma showed inconsistency later on, prediction in non-small cell lung cancer (NSCLC) is more consistent.^[9,10]^ Compared with IrAE that may differ between trials, we suggest treatment-related adverse events (TrAEs) are more generalized and inclusive. A previous study by our fellow colleagues showed that TrAEs are significantly predictive of response of ICB in an older era when ICB was used as monotherapies across cancers.^[11,12]^ In the current study, we have updated the study pool to the very recent (Dec 2019) and have extended inclusion criteria by encompassing recent trials with ICB combination therapy. We aim to validate our hypothesis that frequency of adverse events can predict response to ICB.

## 2 Methods

### 2.1 Search strategy

We searched MEDLINE and Google Scholar (Dec 1, 2012 to Dec 30, 2019) with modification to established criteria^[13]^ using search terms Nivolumab, BMS-936558, Pembrolizumab, MK-3475, Atezolizumab, MPDL3280A, Durvalumab, MEDI4736, Avelumab, MSB0010718C, BMS-936559, Cemiplimab, and REGN2810, and Ipilimumab. Only reports in English language were allowed. Conference proceedings, references of relevant review articles, citations of included studies, and trial cooperative-group websites were hand-searched.

### 2.2 Study selection

Randomized trials of all types of cancer that enrolled at least 10 patients who were not selected for PD-L1 tumor expression, treated with regimen containing anti-PD-1, anti-PD-L1, or anti-CTLA-4 agents and that reported TrAEs, either any or grade 3 to 5 (G3–5) or both, were allowed. Studies that reported IrAEs instead of TrAEs were also allowed. Lines of ICB treatment were not designated as long as reported TrAEs were specified to ICB. Trials that were terminated prematurely due to unexpected toxicity were excluded.

### 2.3 Data extraction

For each included trial, we extracted the trial registration ID, identifier of publication (e.g., DOI), ORR, % of any and G3–5 TrAEs, and number of participants allocated to the ICB arm. As both ORR and TrAEs were descriptive data with percentage, no hazard ratios and or 95% confidence intervals (CIs) were available.

### 2.4 Statistical analysis

ORR was plotted against % of any and G3–5 TrAEs and a linear regression model was fitted. As large differences existed in the current study that encompassed a variety of cancers treated with different types and doses of ICB drugs, and that our primary aim was to observe overall trending of correlation between AE and response, all analyses were performed unweighted by trial size. The Pearson *r*^2^ value of 0.72 or greater was considered a strong correlation, and *r*^2^ from 0.49 to less than 0.72 was considered modest correlation. Subgroup analyses were also carried out in select cancer types that were investigated in more than 5 trials. Graphs were plotted by Plotly (https://chart-studio.plot.ly/) and statistical analyses were run by Prism Graphpad ver 7.

## 3 Results

**Figure 1:**
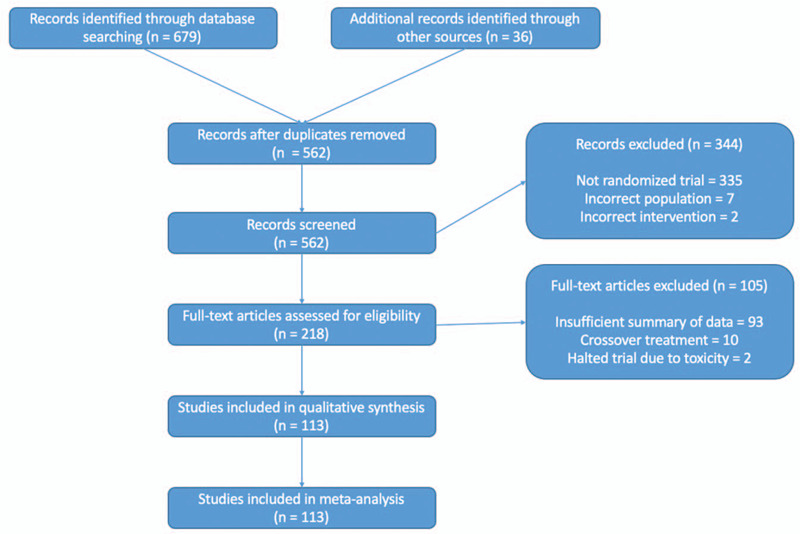
PRISMA (preferred reporting items for systematic reviews and meta-analyses) diagram/flow chart.

We identified 113 eligible studies encompassing 25 types of malignancies that were treated with ICB or ICB-based regimes (Fig. 1 and see Supplemental Table 1 which listed all studies included in the analysis herein). There were a total of 21,504 patients included. Atezolizumab-based regimen was reported in 13 trials, whereas Avelumab-based reported in 10 trials, Durvalumab-based in 6 trials, Ipilimumab monotherapy in 6 trials, Nivolumab-based in 33 trials, Nivolumab plus Ipilimumab in 14 trials, Pembrolizumab-based in 28 trials, and multiple ICB in 1 study.

### 3.1 TrAEs predict ICB response overall

**Figure 2:**
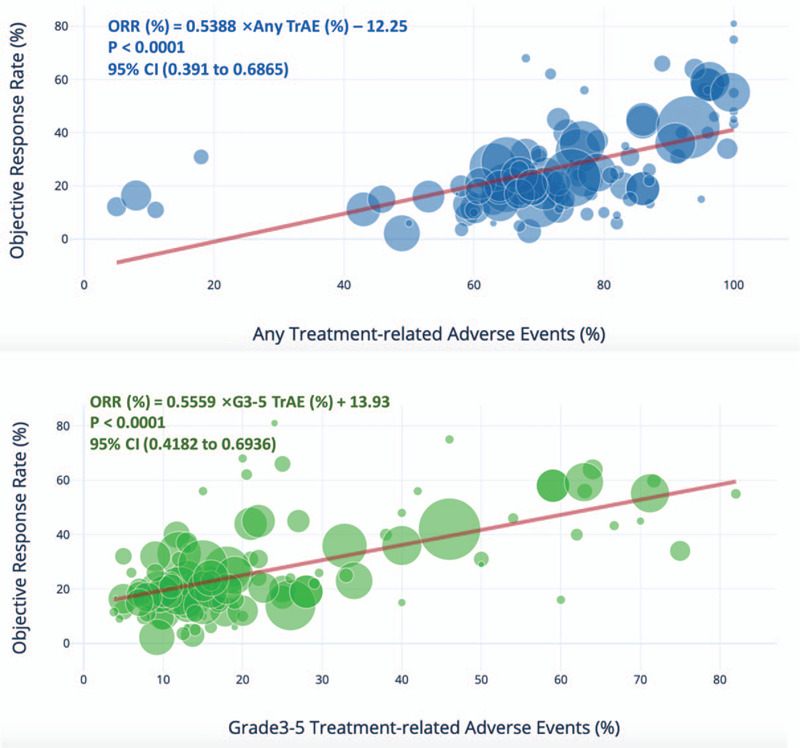
Shown is the treatment-related adverse events (TrAEs) of any and grade 3 to 4 among patients who received immune checkpoint blockade (ICB)-based regimen, as described in published studies for which data regarding the objective response rate are available. Equations were deduced from linear regression. CI stands for confidence interval. Circle size represents trial size which was unweighted in the analysis.

Figure 2 demonstrates ORR against all-grade and G3–5 TrAEs, respectively. A significant linear correlation was observed for any and severe TrAEs, respectively. The correlation coefficient was 0.57 (*r*^2^ = 0.324) for any TrAEs and 0.61 (*r*^2^ = 0.37) for G3–5 TrAEs, indicating that over half of ICB responses could be reflected by any adverse events and ∼60% of responses could be reflected by severe AEs. In general, Hodgkin's lymphoma and Merkel cell carcinoma showed a better response rate that could be predicted by AEs, whereas ovarian cancer, adrenal corticocarcinoma, and uveal melanoma showed a worse response rate that could be predicted by AEs. Major cancer types that were tested in multiple ICB trials like melanoma, renal cell carcinoma, and NSCLC scattered close to the regression line indicating a major effect in generating the correlation.

### 3.2 TrAEs predict ICB response in select cancers

**Figure 3:**
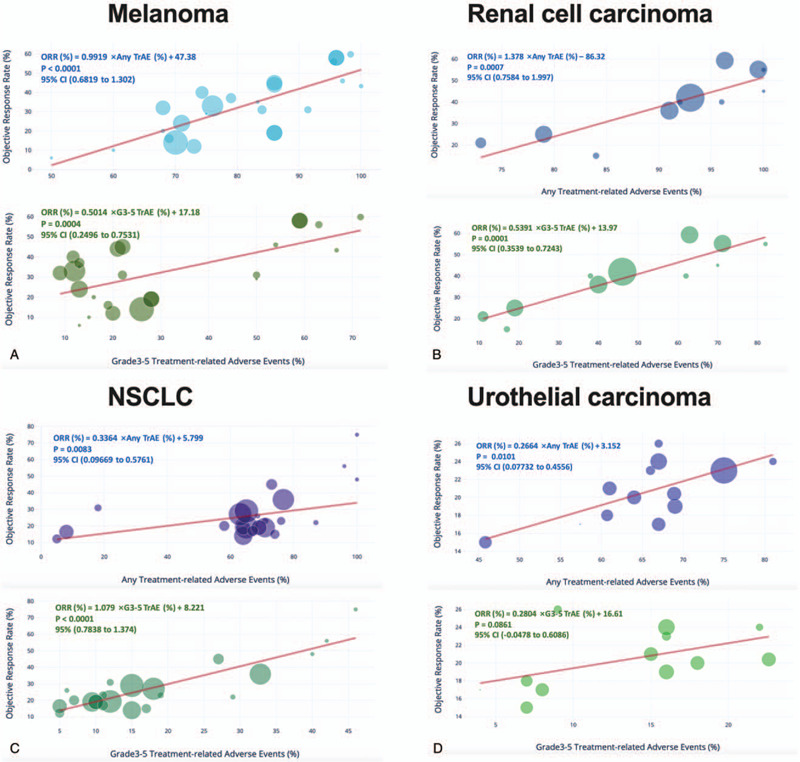
Select cancer types including A) melanoma, B) renal cell carcinoma, C) non-small cell lung cancer (NSCLC), and urothelial cancer were plotted separately for response to ICB against percentage of any and TrAEs. Equations were deduced from linear regression. CI stands for confidence interval. Circle size represents trial size which was unweighted in the analysis.

We therefore further investigated correlations for select cancer types that were tested in more than 5 trials. For melanoma, the correlation coefficient was 0.81 (*r*^2^ = 0.57) for any TrAE and 0.65 (*r*^2^ = 0.42) for G3–5 TrAE (Fig. 3A). For RCC, the correlation coefficient was 0.86 (*r*^2^ = 0.74) for any TrAE and 0.91 (*r*^2^ = 0.83) for G3–5 TrAEs (Fig. 3B). For NSCLC, the correlation coefficient was 0.55 (*r*^2^ = 0.3) for any TrAE and 0.74 (*r*^2^ = 0.86) for G3–5 TrAEs (Fig. 3C). For UC, the correlation coefficient was 0.47 (*r*^2^ = 0.68) for any TrAEs and 0.27 (*r*^2^ = 0.52) for G3–5 TrAEs, yet the correlation was insignificant for severe AEs (Fig. 3D).

### 3.3 Response to Nivolumab and Atezolizumab are predictable by TrAEs

Finally, we explored predictive value of AEs categorized by drug type. As Durvalumab and Ipilimumab monotherapy were solely tested in 6 and 8 trials, respectively, we excluded analyses of those 2 agents. For both Nivolumab and Atezolizumab, both any TrAEs (*P* < .0001 for both Nivolumab and Atezolizumab) and G3–5 TrAEs (*P* < .0001 for Nivolumab and *P* < .0004 for Atezolizumab) were significantly correlated with drug response, respectively. For Pembrolizumab, only any TrAEs were significantly correlated with ORR (*P* < .0101), whereas G3–5 TrAEs were not (*P* = .0861). For Avelumab, neither any (*P* = .1189) nor G3–5 (*P* = .0583) TrAEs was significantly correlated with response.

## 4 Discussion

Emergence of ICB has substantially enhanced efficacy compared with traditional immunotherapy with characteristic adverse events.^[14–16]^ In general, TrAEs associated with ICB consist of inflammation of almost all organs. Integumentary TrAEs are most common, presenting as hives, eczema, vitiligo, etc. Gastrointestinal TrAEs include enterocolitis, pancreatitis, etc. Cardiac TrAEs, specifically fulminant myocarditis is of note the most severe AE which entails a high mortality.^[17]^ Endocrine TrAEs like hyper- or hypo-thyroidism and hypophysitis may require hormone replacement and can be lasting. In general, most TrAEs appear within 2 to 3 months of treatment and are reversible with immunosuppression or steroids, without compromising anti-cancer effect.^[18]^

Although mechanistically speaking, systemic AEs entailed by checkpoint inhibitor has little association with its anti-cancer activity, some recent studies did show correlations between IrAEs and TrAEs with ICB outcome. Downey et al showed that melanoma patients with grade 3 to 4 IrAEs had duration of response more than doubled than those with grade 1 to 2 IrAEs.^[9]^ Sanlorenzo et al showed that melanoma patient developing cutaneous adverse events while on Pembrolizumab had significantly longer progression-free survival (PFS) compared with patients who did not.^[19]^ Indini et al reported IrAEs correlated with improved OS and PFS in patients undergoing anti-PD-1 immunotherapy for metastatic melanoma.^[20]^

For NSCLC, the predictive effect of TrAE appears more robust. Haratani et al showed that NSCLC patients on Nivolumab had significantly longer median overall survival in the presence of IrAE, which were also positively associated with PFS.^[10]^ Toi et al reported in a retrospective NSCLC patient cohort that incidence of any grade IrAE was significantly higher in responders than nonresponders, together with a PFS benefit of ∼9 months.^[21]^ Sato et al showed that Nivolumab treated NSCLC patients with IrAE had significantly higher ORR than those without, with a difference of 56.2%. Also PFS was also significantly longer for patients with IrAEs (HR = 0.1).^[22]^ Teraoka et al reported early IrAEs that occurred within 2 to 6 weeks of commencement of Nivolumab was significantly associated with a ∼5 month longer PFS. Late IrAEs onset also showed a trend toward better PFS.^[23]^ Lisberg et al also reported that TrAEs predict improved clinical outcome in NSCLC patients in Keynote-001 trial at a single center.^[24]^ Interestingly, such survival benefit was also reported in head and neck cancer. Foster et al showed significant association of IrAEs with improved response, PFS, and overall survival for patients with metastatic head and neck cancer receiving anti-PD-1 therapy.^[25]^

Based on those cumulative data, Ou et al investigated association of IrAEs with immune checkpoint inhibitor efficacy in pancancer.^[26]^ They showed that development of pneumonitis and diarrhea was associated with survival outcome of immune checkpoint inhibitors in patients with advanced cancer. In line with previous report by our fellow colleagues,^[12]^ the current updated data on TrAEs incurred by ICB-based therapy showed significant association between ORR and response. We suggest that the association for ORR does not necessary translate to survival. However, current data already provide strong evidence that cautious management of TrAEs can lead to achieving maximum clinical benefit from ICB treatment.

## 5 Conclusion

In the current study, our findings suggest that over half of ICB responses could be reflected by any adverse events and ∼60% of responses could be reflected by severe AEs. Melanoma, RCC, NSCLC, and UC are cancer types that show strong correlations. Further validation is needed in individual trials.

## Author contributions

YS and YC drafted the manuscript, ZZ and DW did statistical analysis, and ZZ oversaw all protocols.

## Supplementary Material

Supplemental Digital Content
